# Supplementary material for: Gender differences in the longitudinal association between obesity, and disability with workplace absenteeism in the Australian working population
Source: PLoS One. 2020 May 27;15(5):e0233512. doi: 10.1371/journal.pone.0233512 (PMC7252611; doi:10.1371/journal.pone.0233512)
Supplement: Attachment — Submitted filename: PONE-D-19-32153_Keramat_editorial.docx [file pone.0233512.s001.docx]

### PLOS ONE

#### Gender differences in the longitudinal association between obesity, disability, and workplace absenteeism in the Australian working population

--Manuscript Draft--

| **Manuscript Number:** | PONE-D-19-32153 |
| --- | --- |
| **Article Type:** | Research Article |
| **Full Title:** | Gender differences in the longitudinal association between obesity, disability, and workplace absenteeism in the Australian working population |
| **Short Title:** | longitudinal association between obesity, disability, and workplace absenteeism |
| **Corresponding Author:** | Syed Afroz Keramat Khulna University Khulna, BANGLADESH |
| **Keywords:** | Obesity; disability; absenteeism; ZINB regression; Australia |
| **Abstract:** | Background: Excess weight can increase absenteeism of workers and can have a negative influence on their productivity. Current evidence on this association are mostly based on cross-sectional data and there is little evidence concerning the longitudinal relationship between obesity, disability and workplace absenteeism. Further, gender differences in this association have often ignored in the existing literature.  Objectives: This study aims to examine gender differences in the longitudinal association between obesity, disability, and absenteeism in the workplace. Methods: Data from twelve waves (2007 to 2018) of the Household, Income and Labour Dynamics in Australia (HILDA) survey were pooled, resulting in 107,669  observations for 19,174 adult employees. The Zero-Inflated Negative Binomial (ZINB) regression model was deployed to investigate the links between obesity, disability, and workplace absenteeism for total sample and stratified by gender.  Results: The findings showed that obese class I (lowest rank of obesity; IRR: 1.39, 95% CI: 1.05-1.84), obese class II (middle rank of obesity; IRR: 2.18, 95% CI: 1.43- 3.33) and workers with a disability (IRR: 2.79, 95% CI: 2.15-3.62) are associated with prolonged workplace absenteeism for the total sample. In addition, the study found that the interaction of overweight and disability (IRR: 1.53, 95% CI: 1.05-2.24) is associated with longer days of absence in the total sample but not found significant in case of female workers. Interestingly, the interaction of obesity and disability was not positively associated with workplace absenteeism independent of gender.  Conclusions: Workplace absenteeism is significantly associated with overweight, obese class I, and obese class II among Australian female workers. An active workplace health promotion program is a necessity for weight management of these workers and thus reduce workplace absenteeism. |
| **Order of Authors:** | Syed Afroz Keramat |
|  | Khorshed Alam |
|  | Jeffrey Gow |
|  | Stuart J. H. Biddle |
| **Opposed Reviewers:** |  |
| **Additional Information:** |  |
| **Question** | **Response** |
| **Financial Disclosure**  Enter a financial disclosure statement that describes the sources of funding for the work included in this submission. Review the [submission guidelines](http://journals.plos.org/plosone/s/submission-guidelines#loc-financial-disclosure-statement) for detailed | This research did not receive any specific grant from funding agencies in the public, commercial or not-for-profit sectors. |

| requirements. View published research articles from [*PLOS ONE*](http://journals.plos.org/plosone/) for specific examples.  This statement is required for submission and **will appear in the published article** if the submission is accepted. Please make sure it is accurate. |  |
| --- | --- |
| **Unfunded studies**  Enter: *The author(s) received no specific funding for this work.*  **Funded studies**  Enter a statement with the following details:   - Initials of the authors who received each award - Grant numbers awarded to each author - The full name of each funder - URL of each funder website - Did the sponsors or funders play any role in the study design, data collection and analysis, decision to publish, or preparation of the manuscript? - **NO** - Include this sentence at the end of your statement: *The funders had no role in study design, data collection and analysis, decision to publish, or preparation of the manuscript.* - **YES** - Specify the role(s) played. |  |
| * typeset |  |
| **Competing Interests**  Use the instructions below to enter a competing interest statement for this submission. On behalf of all authors, disclose any [competing interests](http://journals.plos.org/plosone/s/competing-interests) that could be perceived to bias this work—acknowledging all financial support and any other relevant financial or non- financial competing interests.  This statement **will appear in the published article** if the submission is accepted. Please make sure it is accurate. View published research articles from [*PLOS ONE*](http://journals.plos.org/plosone/) for specific examples. | The authors have declared that no competing interests exist. |

| **NO authors have competing interests**  Enter: *The authors have declared that no competing interests exist.*  **Authors with competing interests**  Enter competing interest details beginning with this statement:  *I have read the journal's policy and the authors of this manuscript have the following competing interests: [insert competing interests here]* |  |
| --- | --- |
| * typeset |  |
| **Ethics Statement**  Enter an ethics statement for this submission. This statement is required if the study involved:   - Human participants - Human specimens or tissue - Vertebrate animals or cephalopods - Vertebrate embryos or tissues - Field research   Write "N/A" if the submission does not require an ethics statement.  General guidance is provided below. Consult the [submission guidelines](http://journals.plos.org/plosone/s/submission-guidelines#loc-guidelines-for-specific-study-types) for detailed instructions. **Make sure that all information entered here is included in the Methods section of the manuscript.** | Ethics approval is not required for the study as it used secondary data from the HILDA survey data set. Researchers working in Australia may use this data set after following certain regulations. There is no identifiable information of individuals in the data. |

| **Format for specific study types**  **Human Subject Research (involving human participants and/or tissue)**   - Give the name of the institutional review board or ethics committee that approved the study - Include the approval number and/or a statement indicating approval of this research - Indicate the form of consent obtained (written/oral) or the reason that consent was not obtained (e.g. the data were analyzed anonymously)   **Animal Research (involving vertebrate animals, embryos or tissues)**   - Provide the name of the Institutional Animal Care and Use Committee (IACUC) or other relevant ethics board that reviewed the study protocol, and indicate whether they approved this research or granted a formal waiver of ethical approval - Include an approval number if one was obtained - If the study involved *non-human primates*, add *additional details* about animal welfare and steps taken to ameliorate suffering - If anesthesia, euthanasia, or any kind of animal sacrifice is part of the study, include briefly which substances and/or methods were applied   **Field Research**  Include the following details if this study involves the collection of plant, animal, or other materials from a natural setting:   - Field permit number - Name of the institution or relevant body that granted permission |  |
| --- | --- |
|  |  |
| **Data Availability**  Authors are required to make all data underlying the findings described fully available, without restriction, and from the time of publication. PLOS allows rare exceptions to address legal and ethical concerns. See the [PLOS Data Policy](http://journals.plos.org/plosone/s/data-availability) and [FAQ](http://journals.plos.org/plosone/s/data-availability#loc-faqs-for-data-policy) for detailed information. | No - some restrictions will apply |

| A Data Availability Statement describing where the data can be found is required at submission. Your answers to this question constitute the Data Availability Statement and **will be published in the article**, if accepted. |  |
| --- | --- |
| **Important:** Stating ‘data available on request from the author’ is not sufficient. If your data are only available upon request, select ‘No’ for the first question and explain your exceptional situation in the text box. |  |
| Do the authors confirm that all data underlying the findings described in their manuscript are fully available without restriction? |  |
| **Describe where the data may be found in full sentences. If you are copying our sample text, replace any instances of XXX with the appropriate details.**   - If the data are **held or will be held in a public repository**, include URLs, accession numbers or DOIs. If this information will only be available after acceptance, indicate this by ticking the box below. For example: *All XXX files are available from the XXX database (accession number(s) XXX, XXX.*). - If the data are all contained **within the manuscript and/or Supporting Information files**, enter the following: *All relevant data are within the manuscript and its Supporting Information files.* - If neither of these applies but you are able to provide **details of access elsewhere**, with or without limitations, please do so. For example:   *Data cannot be shared publicly because of [XXX]. Data are available from the XXX Institutional Data Access / Ethics Committee (contact via XXX) for researchers who meet the criteria for access to confidential data.*  *The data underlying the results presented in the study are available from (include the name of the third party* | The data used for the study were collected from the Melbourne Institute of Applied Economic and Social Research. There are some restrictions on this data and it is not available to the public. Those interested in accessing this data should contact the Melbourne Institute of Applied Economic and Social Research, The University of Melbourne, VIC 3010, Australia. |

| *and contact information or URL).*   - This text is appropriate if the data are owned by a third party and authors do not have permission to share the data.   * typeset |  |
| --- | --- |
| Additional data availability information: |  |

cover letter

**Cover Letter**

19th November 2019 The Editor

PLOS ONE

We are pleased to submit an original research manuscript entitled, “Gender differences in the longitudinal association between obesity, disability, and workplace absenteeism in the Australian working population” for consideration of publication in your journal.

We have chosen this journal because your journal is very well reputed and we think that the subject fits well with this journal. This study aims to examine gender differences in the longitudinal association between obesity, disability, and absenteeism in the workplace. Data from twelve waves (2007 to 2018) of the *Household, Income and Labour Dynamics in Australia* (*HILDA*) survey were pooled, resulting in 107,669 observations for 19,174 adult employees. The Zero-Inflated Negative Binomial (ZINB) regression model was deployed to investigate the links between obesity, disability, and workplace absenteeism for total sample and stratified by gender. The findings showed that obese class I (lowest rank of obesity; IRR: 1.39, 95% CI: 1.05-1.84), obese class II (middle rank of obesity; IRR: 2.18, 95% CI: 1.43-3.33) and workers with a disability (IRR: 2.79, 95% CI: 2.15-3.62) are associated with prolonged workplace absenteeism for the total sample. In addition, the study found that the interaction of overweight and disability (IRR: 1.53, 95% CI: 1.05-2.24) is associated with longer days of absence in the total sample but not found significant in case of female workers. Interestingly, the interaction of obesity and disability was not positively associated with workplace absenteeism independent of gender. Workplace absenteeism is significantly associated with overweight, obese class I, and obese class II among Australian female workers. An active workplace health promotion program is a necessity for weight management of these workers and thus reduce workplace absenteeism.

We declare that we have no potential conflict of interest. All authors have read this manuscript and approved for final submission. The manuscript has not been submitted or published in any other journal. We shall be grateful if you kindly consider publication of this paper in your fast, fair and professional peer review journal “PLOS ONE” so that a wide range of audience can be reached.

Best regards,

Syed Afroz Keramat

Lecturer, Economics Discipline

Khulna University, Khulna-9208, Bangladesh

Email: [syed.afroz@econ.ku.ac.bd](mailto:syed.afroz@econ.ku.ac.bd) ; Phone: + 88 (0) 1718773722

Manuscript	[Click here to access/download;Manuscript;Manuscript.docx](https://www.editorialmanager.com/pone/download.aspx?id=25547950&amp;guid=50d1f96d-48b4-409f-bc0f-bdadff9f3c21&amp;scheme=1)

## 1 Gender differences in the longitudinal association between

2 ***obesity,	disability,	and	workplace	absenteeism	in	the***
3 ***Australian working population***

4

5	Syed Afroz Keramat1, 2, 3*, Khorshed Alam2, 3, Jeff Gow2, 4, Stuart J.H. Biddle^3^

6

7

#### 8 Author’s affiliation:

9 1 Economics Discipline, Social Science School, Khulna University, Khulna-9208, Bangladesh
10 2 School of Commerce, University of Southern Queensland, Toowoomba, QLD 4350, Australia
11 3 Centre for Health, Informatics and Economic Research, University of Southern Queensland,
12 Toowoomba, QLD 4350, Australia
13 4 School of Accounting, Economics, and Finance, University of KwaZulu-Natal, Durban, 4000,
14 South Africa

15

#### 16 ^*^Address of the corresponding author:

17 Economics Discipline, School of Social Science, Khulna University, Khulna, Bangladesh
18 Phone: + 88 (0) 1718-773722, Email: [syed.afroz@econ.ku.ac.bd](mailto:syed.afroz@econ.ku.ac.bd)

19

20

21

22

# 23 Abstract

24 **Background:** Excess weight can increase absenteeism of workers and can have a negative
25 influence on their productivity. Current evidence on this association are mostly based on cross-
26 sectional data and there is little evidence concerning the longitudinal relationship between
27 obesity, disability and workplace absenteeism. Further, gender differences in this association
28 have often ignored in the existing literature.
29 **Objectives:** This study aims to examine gender differences in the longitudinal association
30 between obesity, disability, and absenteeism in the workplace.
31 **Methods:** Data from twelve waves (2007 to 2018) of the Household, Income and Labour
32 Dynamics in Australia (HILDA) survey were pooled, resulting in 107,669 observations for
33 19,174 adult employees. The Zero-Inflated Negative Binomial (ZINB) regression model was
34 deployed to investigate the links between obesity and disability with workplace absenteeism for
35 total sample and stratified by gender. ## Also, please present the obese categories here.
36 **Results:** The findings showed that obese class I (lowest rank of obesity; IRR: 1.39, 95% CI:
37 1.05-1.84), obese class II (middle rank of obesity; IRR: 2.18, 95% CI: 1.43-3.33) and workers
38 with a disability (IRR: 2.79, 95% CI: 2.15-3.62) are associated with prolonged workplace
39 absenteeism for the total sample. In addition, the study found that the interaction of overweight
40 and disability (IRR: 1.53, 95% CI: 1.05-2.24) is associated with longer days of absence in the
41 total sample but not found significant in case of female workers. The interaction
42 of obesity and disability was not positively associated with workplace absenteeism independent
43 of gender. I am wondering what is interesting here? Was it expected that the people with both obesity and disability would have higher workplace absenteeism? Please write it for lay persons so that they don’t need to think.
44 **Conclusions:** Workplace absenteeism is significantly associated with overweight, obese
45 class I, and obese class II among Australian female workers. An active workplace health
46 promotion program is a necessity for weight management of these workers and thus reduce
47 workplace absenteeism.
48 **Keywords:** Obesity, disability, absenteeism, ZINB regression, Australia

49

# 50 Introduction

51 Globally, the prevalence of obesity has almost tripled since 1975 [1]. Worldwide more than
52 650 million adults aged 18 years or over were obese in 2016 [1].
53 ## I would consider to bring statements from the line 75 to show this is a global issue and the literature at global level. You could reduce some literature which are not going to be looked at in the current study.
55 Then narrow down and introduce the Australian concept, what are the problems, what was found was found and the gap. The prevalence of overweight
56 and obesity among Australian adults is 63% and its rising prevalence has became a serious
57 public health concern [2]. The high health and financial burden of overweight and obesity in
58 Australia has been well documented [3]. Excess weight in individuals is responsible for 7% of
59 the total health burden in the country [3]. Overweight and obese adults are more susceptible to
60 develop chronic conditions like cancers, cardiovascular disease, chronic kidney disease,
61 dementia, diabetes, and osteoarthritis [3]. The direct financial cost of obesity to the Australian
62 economy was estimated to be AUD 3.8 billion in 2011-12 [4]. In addition to the direct costs,
63 overweight and obesity have indirect costs in the form of lost productivity (i.e. increased
64 absenteeism and presenteeism). In 2011-12, the indirect cost of obesity was estimated to be
65 AUD 4.8 billion to the Australian economy [4].

63

64 Absenteeism in the Australian workplace has risen by 7% since 2010 [5]. Approximately
65 ninety-two million workdays are lost annually with the annual cost in the form of lost
66 productivity is estimated to be AUD 33 billion [5]. This is up to 8% of the total payroll costs
67 to Australian companies [5]. The main reasons for employees’ absence are poor health and
68 fitness [6], illness (flu, headache, and gastro), family responsibilities, mental issues, and
69 alcohol/drug-related issues [5]. Employees who are absent from the workplace due to personal
70 illness or injury include obese individuals who take longer leave periods compared with non-
71 obese individuals [7]. According to the NHS survey, over 4 million workdays were lost from
72 Australian workplaces in 2001 due to obesity [7]. This evidence suggests that there might be
73 an association between weight status and absenteeism for the Australian working population.
74 A number of studies have examined the link between obesity and absenteeism using a cross-
75 sectional design [8–17]. Studies conducted on US workers provided evidence that obese
76 employees were more likely to be absent from the workplace compared with their healthy
77 weight counterparts [8,11,17]. In addition, a study conducted on multinational manufacturing
78 workplaces in Ireland found that obese employees were 72% more prone to be absent [9].
79 A recent study on the working population in the Netherlands revealed that obese workers took
80 14 days of extra leave compared to their lower weight counterparts [12]. Furthermore, a study
81 of Belgian workers also provides evidence that obesity is positively associated with high rates
82 of sickness absence in excess of 10 days [16]. Similar results have been found in a British study
83 where the authors claimed that obese individuals were absent for four extra days per year
84 compared with workers of healthy weight [10]. Another study from Britain also revealed that
85 overweight and obesity were correlated with both short and long spells of sickness absence for
86 both male and female workers [13]. However, a study in Germany did not find evidence that
87 overweight men took more sick leave days compared with lower weight workers [15].

88

89 A few studies have also examined the longitudinal association between workplace obesity and
90 absenteeism [18–21]. A prospective study among middle-aged employees in Finland revealed
91 that stable obesity and weight gain in the follow-up period increased the risk of prolonged
92 sickness absence [21]. Two US-based longitudinal studies also provided evidence that obesity
93 is positively associated with absenteeism in the workplace [19,20]. A recent study in Germany
94 examined the gender differences in the longitudinal association between obesity and
95 absenteeism from work and claimed that obesity was positively associated with long-term
96 absenteeism in women but not in men [18].
97 One of the main limitations of past studies that explore the cross-sectional association between
98 obesity and absenteeism is that causality could not be inferred from such observational data
99 [22]. A few studies that have attempted to identify the longitudinal relationship have mostly
100 been based in the US or European countries. Evidence on the longitudinal relationship between
101 obesity and workers' absenteeism from the Australian perspective is still lacking. Moreover,
102 no study to date has explored the association between the interaction of obesity and disability
103 with workplace absenteeism. Additionally, very few studies have investigated gender
104 differences in the longitudinal association between obesity and workplace absenteeism. The
105 present study fills this void in the literature by examining whether obesity are associated with
106 prolonged absenteeism. Moreover, the present study tests the association between interaction
107 of obesity and disability with absenteeism will be examined. To attain the objectives, the
108 present study will address the research question: does a longitudinal association exist between
109 obesity, disability and absenteeism in the workplace?
110 Excessive bodyweight of workers should be a major concern to businesses as there might be a
111 positive association between workplace absenteeism and obesity and thus extra cost to
112 companies. The present study will offer evidence on the longitudinal links between obesity,
113 disability, and workplace absenteeism. The results of the study might be used by policymakers
114 and organizations for the development and implementation of workplace health promotion
115 programs to tackle excessive weight problems of the workers.

# 119 Data and Methods

### 120 Sample

121 The present study used the individual person dataset from the Household, Income and Labour
122 Dynamics in Australia (HILDA) survey. This is a large-scale nationally representative panel
123 survey of Australian households that collects data on family, wealth, health, education, and
124 labour market dynamics [23]. This household panel survey is similar to the Panel Study of
125 Income Dynamics (PSID) in the US, the British Household Panel Survey (BHPS), and the
126 German Socio-Economic Panel (SOEP). The HILDA survey commenced in 2001 and since
127 then has been conducted annually following the University of Melbourne’s ethical guidelines.
128 It collects detailed information from household members aged 15 years and over using a
129 combination of personal face-to-face interviews by trained interviewers and self-completed
130 questionnaires. The survey uses a multi-stage sampling approach, sampling within households
131 within a particular administrative area. Detailed information about the study design has been
132 described elsewhere [23].
133 This study utilized twelve recent waves (waves 6 to 17) from the HILDA dataset. The main
134 reason for choosing the most recent 12 waves of the survey (2007-2018) is that data on Body
135 Mass Index (BMI) are available only in these waves. The inclusion criteria of the study are
136 prospective subjects aged 15-64 years and employed at each wave. Subjects with missing
137 information on days absent from the workplace in the last 12 months were excluded (n = 2360
138 observations). Further, pregnant employees were excluded (n = 8631 observations) from the
139 subsample analyses. After employing exclusion criteria and excluding missing data, the
140 unbalanced panel consists of 107,669 subjects for 19,174 adult employees.

### 142 Measures

143 **Outcome variable**
144 The main outcome variable of the study is days absent from work on paid workers'
145 compensation in the last twelve months**.** It is a derived variable and was constructed using the
146 variable work schedule to determine the number of days absent from workplace.

### 147 Exposure variables

148 The main variables of interest in the present study are BMI and disability. BMI is calculated
149 using self-reported height and weight following the formula weight (in kilograms) divided by
150 height (in meters squared). This study categorized BMI into four groups following the World
151 Health Organization (WHO) guidelines: <18.50 (underweight), 18.50-24.99 (normal/healthy

152	weight), 25.00-29.99 (overweight), 30.00-34.99 (obese class I), 35.00-39.99 (obese class II),

153 and ≥40.00 (obese class III) [1]. Underweight is not a topic of interest in the current study. As
154 a result, this study merged two BMI categories (underweight with healthy weight) and form a
155 new category, <25 BMI, following relevant studies conducted in Australia and The Netherlands
156 [14,24] to conduct the regression analysis.
157 Disability of an adult used in the HILDA survey was based on the guidelines of the
158 International Classification of Functioning, Disability, and Health (ICF) under the WHO
159 framework [25]. Participants were asked if they have any ‘disability, impairment or disability
160 that restricts them in everyday activities, and has lasted or are likely to last, for 6 months or
161 more’ [26]. Responses were coded in binary form (yes or no). Participants who answered ‘yes’
162 were counted as an adult with disability.

### 163 Other covariates

164 Potential covariates of absenteeism in the workplace were selected from the existing literature.
165 This study includes age (young worker: 15-35 years, middle-aged worker: 36-55 years, and
166 older worker: 56-64 years) [8,10,11,18], sex (male and female) [10,11,19], marital status (non-
167 cohabitating and married) [18], and education (year 12 or below, professional qualification,
168 and university qualification) [8,11] as socio-demographic characteristics.
169 The present study also included eleven measures of job-related characteristics that includes
170 firm size (small, medium, and large) [27], employment contract (permanent, fixed-term and
171 casual) [27,28], tenure with current employer (1-5 years, and 6 or more years) [27], hours per
172 week usually worked (<35, 35-40, and >40 hours a week) [29], work schedule (day work and
173 shift work) [27,29], job type (non-manual and manual workers) [11,29], supervisory
174 responsibility (yes and no), paid holiday leave (yes and no), paid sick leave (yes and no)
175 [20,29], union membership (yes and no) [27,28], and overall job satisfaction (measured in an
176 ordinal scale which ranged from 0 [totally dissatisfied] to 10 [totally satisfied]) [29].
177 ## One of the main focus of this study is the Gender difference (in the Title). However, this is difficult to find where it was first reported. It is possibly first time in line 261. It could be introduced earlier, possibly after the outcome variable.

### 178 Methods

179 To explore the factors associated with workplace absenteeism, the present study followed the
180 conceptual framework of Hafner et al. [30]. Accordingly, factors of workplace productivity
181 (absenteeism and presenteeism) are broadly categorized into three groups and can be expressed
182 as follows.
183 Y_i_ = *f* ( j, p, h)
184 In the function, Y_i_ refers to workplace productivity (i.e. absenteeism), j refers to job-related
185 factors (i.e. work demands), p refers to personal factors (i.e. lifestyle factors), and h refers to
186 health and physical factors (i.e. long-term health conditions).
187 The outcome variable, workplace absenteeism, is a count variable where all the values are non-
188 negative integer numbers including zero. The negative binomial model is appropriate to
189 estimate the association between covariates and the outcome variable when the outcome
190 variable is a count variable and overdispersed [31]. In the present study, the number of zeros
191 in the outcome variable is excessive. Among these zeros, there are two kinds of zero values.
192 First, there are some certain zeros because employees may not be absent in the workplace due
193 to work restrictions. Second, there might exist zeros for employees who were not absent in the
194 workplace but could be absent due to sickness or other conditions. Hence, the number of zeros
195 might be inflated in the outcome variable due to certain zeroes. The standard negative binomial
196 regression model cannot differentiate between these two processes when they arrive at a zero
197 value in the outcome variable [32]. However, the Zero-Inflated Negative Binomial (ZINB)
198 model can handle these two distinct data generation processes [32]. The ZINB model fits a
199 logistic regression model to predict the excess zeros in the dependent variable (absenteeism)
200 and then fits the negative binomial regression model to get a count of the number of days absent
201 for non-excess zeros [33]. Given this, the current study followed standard practice and
202 employed the ZINB regression model to estimate the longitudinal association between obesity,
203 disability and workplace absenteeism. The study results are demonstrated in the form of
204 incidence rate ratio (IRR) for each variable.
205 This study initially employed a bivariate model to assess the relationship between obesity and
206 absenteeism (model 1). Subsequently, the present study explores this longitudinal association
207 after controlling for disability and its interaction with obesity (model 2), socio-demographic
208 characteristics (model 3), and job-related characteristics (model 4). In order to explore the
209 gender differences in the association, the present study conducted a separate analysis for males
210 and females along with overall population estimates. Stata 14 windows version was used for
211 all statistical analyses. This study set a p-value at <0.05 level for statistical significance.

### 212 Ethics approval

213 Ethics approval is not required for the study as it used secondary data from the HILDA survey
214 data set. Researchers working in Australia may use this data set after following certain
215 regulations. There is no identifiable information of individuals in the data.

# 217 Results

### 218 Descriptive characteristics of the study sample

\## Important: 1. Some results are discussed in the results section which should go to the discussion. Some statements are related to materials and methods are in the results section. Some results are presented with too details whereas those are presented in Tables and Figures. I would consider to present the key findings and refer to the Tables/Figures.

2. Absenteeism is too skewed. Please present with median and range.

3. Please report what are the models 1, 2, 3 and 4 are (covariate adjusted) and present only Model 1 and Model 2. If there are no changes in models after additional covariate adjustment, no points to show the Models. It can be said in the text that this………….was done but the model did not change. Even, Model 1 can be presented in the text and Model 2 can be presented in Table 2 for Total and by Gender by combining Table 2, 3, and 4.

This will give side by side comparison without loosing any information.

219 Descriptive statistics in the form of frequency (n) and percentages (%) with 95% confidence
220 intervals (CI) were used to describe the weight status of the study participants. Table 1 shows
221 the pooled characteristics of the employees in terms of overweight, obesity, disability,
222 absenteeism, socio-demographic characteristics, and job-related characteristics. Study
223 participants of the present study were evenly split by sex (51% male), predominantly middle-
224 aged (45%) and partnered (60 %), and 38% had completed year 12 or less education. Table 1
225 shows that workers were mostly from small firms (44%), had permanent employment contracts
226 (66%), worked 35-40 hours a week (36%), were day workers (75%), undertook non-manual
227 jobs (51%), did not have supervisory responsibilities (54%), enjoyed paid holiday leave (72%),
228 paid sick leave (73%), and did not belong to a union or employee association (77%). The mean
229 job satisfaction level of the workers was 7.64 on a 10-point scale.
230 Around 52% were either normal weight or underweight (<25 BMI), 29% were overweight, and
231 19% (19,998 workers) were obese. An estimated 16% of Australian workers have a disability.
232 The average number of absent days of workers is 0.70, although the standard deviation (8.70)
233 is very high. A higher value of the standard deviation over mean absent days indicates an
234 overdispersion of the variable. The mean absent days are quite low due to the presence of
235 excessive zeros in the absenteeism variable.
236 Insert Table 1 here
237 Figure 1 demonstrates the average absent days of the workers by weight status (normal weight,
238 overweight, obese class I, obese class II, and obese class III). Average absenteeism is
239 significantly higher among overweight and obese employees compared with lower weight
240 employees. Figure 1 reveals that the average number of missed days is highest among the
241 morbidly obese (obese class III) workers (1.76), followed by obese class 2 workers (1.34 days).
242 The mean number of missed days of workers with BMI<25 is only 0.50 days.
243 Insert Figure 1 here

### 244 Factors associated with workplace absenteeism

245 Estimates of the longitudinal association between obesity, disability and absenteeism in the
246 workplace after controlling for socio-demographic and job-related characteristics are presented
247 in Table 2. The results show a set of significant links between overweight, obesity, and
248 absenteeism in the bivariate model (model 1). The inclusion of control variables like disability,
249 the interaction of weight status and disability, socio-demographic factors and job-related
250 factors produced similar results in the final model (model 4). The results show that obese class
251 I and II were significantly associated with absenteeism but not with overweight and obese class
252 III. The findings indicate that obese class I and class II workers were 1.39 (IRR: 1.39, 95% CI:
253 1.05-1.84) and 2.18 (IRR: 2.18, 95% CI: 1.43-3.33) times more likely to be absent from the
254 workplace compared with their lower weight counterparts, respectively. Similarly, the number
255 of days absent from the workplace among workers with disability was 2.79 (IRR: 2.79, 95%
256 CI: 2.15-3.62) times higher compared with workers without disability. Interestingly, the results
257 show that the number of days absent in the workplace is 1.53 times (IRR: 1.54, 95% CI: 1.05-
258 2.24) higher among overweight workers with a disability. However, this study does not find a
259 positive association between the interaction of obesity and disability and prolonged
260 absenteeism.
261 Insert Table 2 here
262 The present study also explored the relationship between obesity, disability and absenteeism
263 by gender. Table 3 reports the results obtained from bivariate (model 1) and multivariate
264 models (models 2 to 4) for male workers. Table 2 shows that male workers’ weight status was
265 not associated with absenteeism in the bivariate model (model 1). However, adding all the
266 controls in the final model (model 4) it is observed that obese class II was positively associated
267 with absenteeism. The study revealed that male obese class II workers were 2.02 (IRR: 2.02,
268 95% CI: 1.01-4.06) times more prone to be absent from the workplace. The study also found
269 evidence that disability (IRR: 2.55, 95% CI: 1.70-3.83), and the interaction between overweight
270 and disability (IRR: 2.58, 95% CI: 1.42-4.71) increased male workers likelihood of being
271 absent from the workplace. The study findings also suggests that there is no significant
272 association between interactions of obesity and disability and absenteeism.
273 Insert Table 3 here
274 Table 4 displays the association between female workers’ weight status, disability and
275 absenteeism. Overweight and obesity were found to be statistically significant with
276 absenteeism in the bivariate model (model 1). Adjusting for potential confounders produced
277 similar results in the final model (model 4); overweight, obese class I, and II were found to be
278 significantly associated with absenteeism. The results showed that female workers belonging
279 to overweight, obese class I and II were 1.72 (IRR: 1.72, 95% CI: 1.18-2.51), 1.77 (IRR: 1.77,

279	95% CI: 1.15-2.73), and 2.87 (IRR: 2.87, 95% CI: 1.60-5.13) times, respectively, more likely

280 be absent from the workplace compared with female workers in lower BMI groups. Like male
281 workers, disability (IRR: 3.08, 95% CI: 2.15-4.41) was found to be statistically significant for
282 female workers. However, the present study did not find evidence that the interaction between
283 overweight, obesity and disability was associated with female workers’ absenteeism.
284 Insert Table 4 here

# 285 Discussion

286 The purpose of the present study was to assess associations over time between obesity,
287 disability and workplace absenteeism in Australian workers, and to test for gender differences
288 in such associations. This study pooled 12 waves of data from the nationally representative
289 sample	of	the	HILDA	survey.	Controlling	for	socio-demographic	and	workplace
290 characteristics, ZINB regression analysis showed that obesity and disability are associated with
291 prolonged absenteeism for the entire participants. A number of observational studies also
292 confirm that obese workers tend to have a higher number of work absences [8–13,16]. In
293 addition to cross-sectional study findings in the literature, a recent study has also confirmed a
294 longitudinal association between obesity and workplace absenteeism [20]. Further, in support
295 of the present study findings, an existing longitudinal study found that obesity was associated
296 with extra sick leave days and long-term workplace absenteeism in women but not in male
297 workers [18]. It was already well documented that obesity is a major risk factor for a number
298 of chronic diseases [1]. Obese workers missed more days of work due to personal illness or
299 injury compared with non-obese workers [7].
300 The study results also revealed that the interaction of overweight and disability increases the
301 likelihood of absent days for male workers but not for female workers. This finding is in line
302 with a study from the Netherlands where the authors found that the interaction of overweight
303 and distress is positively associated with long-term sickness absence [14]. The association
304 between the interaction of overweight and disability with higher absenteeism might be
305 explained by the fact that comorbidities lead to a higher number of absent days [12,14].
306 In line with the existing evidence, the current study results revealed that there is no longitudinal
307 association between overweight and more absence days in the total sample [10,34]. However,
308 the results show that overweight and absenteeism are positively associated in the long-run
309 among female workers. This finding is also supported by an observational study that found
310 overweight people are more likely to be absent from the workplace [16]. These dissimilar
311 findings might be due to differences in population characteristics, study settings, and
312 definitions of absenteeism [14].
313 In addition, the present study results do not find evidence that the interaction between obesity
314 and disability are positively associated with absenteeism for the total sample. This finding is
315 similar to a US-based longitudinal study where the authors did not find evidence of an
316 association between the	interactions	of obesity with	four chronic health conditions of
317 hypertension, coronary heart disease, other heart diseases, and stroke, and absenteeism [20].
318 Knowledge of the longitudinal association between obesity and absenteeism is important to
319 companies and policymakers to take measures to reduce the rate of absenteeism in the
320 workplace [18]. From the viewpoint of public policy, the results of this longitudinal study will
321 help policymakers to have a more comprehensive understanding of absenteeism in the
322 workplace due to excessive weight. The results suggest that companies should focus on an
323 integrated lifestyle approach for weight management of their workers by using multiple
324 intervention strategies. Companies should create a supportive environment by enabling
325 physical infrastructure and workplace culture to encourage a healthy lifestyle. For example,
326 companies may offer healthy catering services, establish gym and activity centres for physical
327 activity, establish on-site bicycle storage, and provide walking maps and routes. The
328 effectiveness of workplace-targeted interventions is currently unclear. However, there is
329 evidence that the absenteeism rate is low among workers who perform physical activities
330 regularly [35,36].
332 The study contributes to the existing literature in a number of ways. First, this is the first study
333 on the association between obesity, disability and absenteeism in the Australian context.
334 Second, the present study pooled a nationally representative longitudinal sample of 107,669
335 observations for 19,174 workers where participants were observed for 12 years to offer precise
336 estimates on the association. Third, the study incorporated a large number of workplace
337 characteristics as control variables including less investigated factors (work schedule, job type,
338 paid and sick leave arrangement) which are associated with absenteeism. Fourth, this is the
339 first study that examines the effect of the interactions between obesity and disability on
340 absenteeism.
341 The present study has some limitations. First, the unbalanced longitudinal design of the study
342 draws longitudinal associations but it is not possible to discern the causal effect of obesity,
343 disability and their interactions on workplace absenteeism. Second, the study findings might
344 be vulnerable to bias, as data on BMI and absenteeism are self-reported. Self-reported bias is
345 high among overweight and obese adults, as they tend to overestimate their height and
346 underestimate their weight [37,38]. The authors call for a well-designed clinical study or a
347 strongly balanced prospective longitudinal study that can draw causal inferences on the
348 association between obesity, disability, and absenteeism.

# 349 Conclusions

350 This study aimed to examine the gender differences in the longitudinal association between
351 obesity, disability and absenteeism. Using the ZINB regression technique, the present study
352 found evidence of an association and compared the results with existing evidence. Robust
353 evidence was found that workplace absenteeism is higher among obese and workers with a
354 disability. It was also revealed that interactions of overweight and disability are associated with
355 workers' absenteeism. However, this study did not find evidence that interactions of obesity
356 and disability are associated with prolonged absenteeism. Our findings are important evidence
357 in the consideration of workplace health promotion policies. Implementation of workplace
358 health promotion programs to treat workers excess weight might be an effective tool to lower
359 the rate of absenteeism.

#### 360 Abbreviation

| 361 | AUD | Australian Dollar |
| --- | --- | --- |
| 362 | BMI | Body Mass Index |

| 363 | HILDA | Household, Income and Labour Dynamics in Australia survey |
| --- | --- | --- |
| 364 | IRR | Incidence Rate Ratio |
| 365 | WHO | World Health Organization |
| 366 | ZINB | Zero-Inflated Negative Binomial |

367 **Acknowledgments**
368 The authors would like to thank the Melbourne Institute of Applied Economic and Social
369 Research for providing the HILDA data set.
370 **Author’s contributions:** SAK initiated the study, conducted the data analysis and drafted
371 the manuscript. KA, JG and SB offered advice, critical comments, and edit the draft
372 manuscript. All the authors read and approved the final manuscript.

#### 373 Conflict of Interest

374 The authors declare that they have no conflicts of interest.

#### 375 Availability of data and materials

376 The data used for the study were collected from the Melbourne Institute of Applied Economic
377 and Social Research. There are some restrictions on this data and it is not available to the public.
378 Those interested in accessing this data should contact the Melbourne Institute of Applied
379 Economic and Social Research, The University of Melbourne, VIC 3010, Australia.

#### 380 Funding

381 This research did not receive any specific grant from funding agencies in the public,
382 commercial or not-for-profit sectors.

# 386 References

387 1.	World Health Organization (WHO). Obesity and overweight [Internet]. 2018 [cited 7
388 May 2019]. Available: htt[ps://www.who.int/news](http://www.who.int/news-room/fact-sheets/detail/obesity-and-)-[room/fact-sheets/detail/obesity-and-](http://www.who.int/news-room/fact-sheets/detail/obesity-and-)
389 overweight
390 2.	AIHW. A picture of overweight and obesity in Australia 2017. Cat. no.PHE 216.
391 Canberra: AIHW; 2017.
392 3.	AIHW. Impact of overweight and obesity as a risk factor for chronic conditions:
393 Australian Burden of Disease Study. Australian Burden of Disease Study series no. 11.
394 Cat. no. BOD 12. Canberra: AIHW; 2017.
395 4.	PwC Australia. Weighing the cost of obesity: a case for action. Australia: PwC Australia;

396	2015.

397 5.	DHS. Absence Management & Wellbeing Survey Report. NSW: Direct Health
398 Solutions; 2016.
399 6.	Wee LH, Yeap LLL, Chan CMH, Wong JE, Jamil NA, Swarna Nantha Y, et al.
400 Anteceding factors predicting absenteeism and presenteeism in urban area in Malaysia.

401	BMC Public Health. 2019;19: 1–12. doi:10.1186/s12889-019-6860-8

402	7.	AIHW.	Obesity	and	Workplace	Absenteeism	Among	Older	Australians.

403	Canberra:AIHW; 2005.

404	8.	Poston WSC, Jitnarin N, Haddock CK, Jahnke SA, Tuley BC. Obesity and injury-related

405	absenteeism in a population-based firefighter cohort. Obesity. 2011;19: 2076–2081.

406	doi:10.1038/oby.2011.147

407	9.	Fitzgerald S, Kirby A, Murphy A, Geaney F. Obesity, diet quality and absenteeism in a

408	working	population.	Public	Health	Nutr.	2016;19:	3287–3295.

409	doi:10.1017/S1368980016001269

410	10.	Harvey SB, Glozier N, Carlton O, Mykletun A, Henderson M, Hotopf M, et al. Obesity

411	and sickness absence: Results from the CHAP study. Occup Med (Chic Ill). 2010;60:

412	362–368. doi:10.1093/occmed/kqq031

413	11.	Frone MR. Obesity and absenteeism among U.S. workers: Do physical health and

414	mental health explain the relation? J Workplace Behav Health. 2007;22: 65–79.

415	doi:10.1080/15555240802157403

416	12.	Jans MP, Van Den Heuvel SG, Hildebrandt VH, Bongers PM. Overweight and obesity

417	as predictors of absenteeism in the working population of the Netherlands. J Occup

418	Environ Med. 2007;49: 975–980. doi:10.1097/JOM.0b013e31814b2eb7

419	13.	Ferrie JE, Head J, Shipley MJ, Vahtera J, Marmot MG, Kivimäki M. BMI, obesity, and

420	sickness	absence	in	the	whitehall	II	study.	Obesity.	2007;15:	1554–1564.

421	doi:10.1038/oby.2007.184

422	14.	Nigatu YT, Roelen CAM, Reijneveld SA, Bültmann U. Overweight and distress have a

423	joint association with long-term sickness absence among Dutch employees. J Occup

424	Environ Med. 2015;57: 52–57. doi:10.1097/JOM.0000000000000273

425	15.	Lehnert T, Stuhldreher N, Streltchenia P, Riedel-Heller SG, König HH. Sick leave days

426	and costs associated with overweight and obesity in germany. J Occup Environ Med.

427	2014;56: 20–27. doi:10.1097/JOM.0000000000000065

428	16. Janssens H, Clays E, Kittel F, De Bacquer D, Casini A, Braeckman L. The association

429	between body mass index class, sickness absence, and presenteeism. J Occup Environ

430	Med. 2012;54: 604–609. doi:10.1097/JOM.0b013e31824b2133

431	17. Tucker LA, Friedman GM. Obesity and absenteeism: An epidemiologic study of 10,825 432	employed adults. Am J Heal Promot. 1998;12: 202–207. doi:10.4278/0890-1171- 433	12.3.202

434	18. Reber KC, König HH, Hajek A. Obesity and sickness absence: Results from a

435	longitudinal nationally representative sample from Germany. BMJ Open. 2018;8: 1–8.

436	doi:10.1136/bmjopen-2017-019839

437	19. Vanwormer JJ, Linde JA, Harnack LJ, Stovitz SD, Jeffery RW. Weight change and

438	workplace absenteeism in the healthworks study. Obes Facts. 2012;5: 745–752.

439	doi:10.1159/000345119

440	20. Howard JT, Potter LB. An assessment of the relationships between overweight, obesity,

441	related chronic health conditions and worker absenteeism. Obes Res Clin Pract. Asia

442	Oceania	Assoc. for	the	Study	of	Obesity; 2014;8: e1–e15.

443	doi:10.1016/j.orcp.2012.09.002

444	21. Roos E, Laaksonen M, Rahkonen O, Lahelma E, Lallukka T. Weight change and

445	sickness absence - A prospective study among middle-aged employees. Eur J Public

446	Health. 2015;25: 263–267. doi:10.1093/eurpub/cku087

447	22. Park S, Pan L, Lankford T. Relationship between employment characteristics and

448	obesity among	employed	U.S. adults. Am	J	Heal Promot. 2014;28:	389–396.

449	doi:10.4278/ajhp.130207-QUAN-64

450 23. Freidin, S., Watson, N. and Wooden M. Household, Income and Labour Dynamics in

451	Australia (HILDA) Survey: Wave 1. Melbourne; 2002.

452	24. Au N, Hollingsworth B. Employment patterns and changes in body weight among young

453	women.	Prev	Med	(Baltim).	Elsevier	Inc.;	2011;52:	310–316.

454	doi:10.1016/j.ypmed.2011.03.006

455	25. LaMontagne AD, Krnjacki L, Milner A, Butterworth P, Kavanagh A. Psychosocial job

456	quality in a national sample of working Australians: A comparison of persons working

457	with versus without disability. SSM - Popul Heal. Elsevier; 2016;2: 175–181.

458	doi:10.1016/j.ssmph.2016.03.001

459	26. Melbourne Institute of Applied Economic and Social Research. Household, Income and

460	Labour Dynamics in Australia (HILDA) Survey: Wave 17 Restricted Release Marked

461	Up Questionnaires. Melbourne, Australia; 2018.

462	27. Bubonya M, Cobb-Clark DA, Wooden M. Mental health and productivity at work: Does

463	what you do matter? Labour Econ. 2017;46: 150–165. doi:10.1016/j.labeco.2017.05.001

464	28. Asay GRB, Roy K, Lang JE, Payne RL, Howard DH. Absenteeism and employer costs

465	associated with chronic diseases and health risk factors in the US workforce. Prev

466	Chronic Dis. 2016;13: 1–11. doi:10.5888/pcd13.150503

467	29. Magee CA, Caputi P, Lee JK. Distinct longitudinal patterns of absenteeism and their

468	antecedents in full-time australian employees. J Occup Health Psychol. 2016;21: 24–36.

doi:10.1037/a0039138

| 469 |  |
| --- | --- |
| 470 | 30. |
| 471 |  |
| 472 | 31. |
| 473 |  |
| 474 |  |
| 475 | 32. |
| 476 |  |
| 477 |  |
| 478 | 33. |
| 479 |  |
| 480 | 34. |
| 481 |  |
| 482 |  |
| 483 | 35. |
| 484 |  |
| 485 |  |
| 486 | 36. |
| 487 |  |
| 488 |  |
| 489 | 37. |
| 490 |  |
| 491 |  |
| 492 | 38. |
| 493 |  |
| 494 |  |
| 495 |  |
| 496 |  |
| 497 |  |
| 498 |  |
| 499 |  |
| 500 |  |
| 501 |  |
| 502 |  |
| 503 |  |
| 504 |  |
| 505 |  |

Hafner, Marco, Christian Van Stolk, Catherine Saunders, Joachim Krapels and BB. Health, wellbeing and productivity in the workplace. RAND EUROPE; 2015.

Trindade DDB, Ospina R, Amorim LD. Choosing the right strategy to model longitudinal count data in epidemiology: An application with CD4 cell counts. Epidemiol Biostat Public Heal. 2015;12: 1–11. doi:10.2427/11520

UCLA. Zero-inflated negative binomial regression [Internet]. 2019 [cited 4 Oct 2019]. Available: https://stats.idre.ucla.edu/stata/output/zero-inflated-negative-binomial- regression/

UCLA. zero-inflated negative binomial regression. [Internet]. 2019 [cited 5 Oct 2019]. Available: https://stats.idre.ucla.edu/r/dae/zinb/

Van Duijvenbode DC, Hoozemans MJM, Van Poppel MNM, Proper KI. The relationship between overweight and obesity, and sick leave: A systematic review. Int J Obes. 2009;33: 807–816. doi:10.1038/ijo.2009.121

van den Heuvel SG, Boshuizen HC, Hildebrandt VH, Blatter BM, Ariëns GA, Bongers PM. Effect of sporting activity on absenteeism in a working population. Br J Sports Med. 2005;39: 5–9. doi:10.1136/bjsm.2004.013052

van Amelsvoort LGPM, Spigt MG, Swaen GMH, Kant Ij. Leisure time physical activity and sickness absenteeism; a prospective study. Occup Med (Chic Ill). 2006;56: 210–

212. doi:10.1093/occmed/kqj026

Maukonen M, Männistö S, Tolonen H. A comparison of measured versus self-reported anthropometrics for assessing obesity in adults: a literature review. Scand J Public Health. 2018;46: 565–579. doi:10.1177/1403494818761971

Gorber SC, Tremblay M, Moher D, Gorber B. A comparison of direct vs. self-report measures for assessing height, weight and body mass index: A systematic review. Obes Rev. 2007;8: 307–326. doi:10.1111/j.1467-789X.2007.00347.x

506	**Figure legend**

507	Fig 1. Average missed days at the workplace by workers’ weight status

508

509

Figure	[Click here to access/download;Figure;figure.jpg](https://www.editorialmanager.com/pone/download.aspx?id=25547567&amp;guid=05e41b58-977d-4ea3-b981-4097447fd3ac&amp;scheme=1)

table

[Click here to access/download](https://www.editorialmanager.com/pone/download.aspx?id=25547566&amp;guid=4785a7dc-d978-4e52-8425-2775e12987dc&amp;scheme=1)

[**Supporting Information** Table.docx](https://www.editorialmanager.com/pone/download.aspx?id=25547566&amp;guid=4785a7dc-d978-4e52-8425-2775e12987dc&amp;scheme=1)
