# Supplementary material for: Gender differences in the longitudinal association between obesity, and disability with workplace absenteeism in the Australian working population
Source: PLoS One. 2020 May 27;15(5):e0233512. doi: 10.1371/journal.pone.0233512 (PMC7252611; doi:10.1371/journal.pone.0233512)
Supplement: Attachment — Submitted filename: R2R.docx [file pone.0233512.s002.docx]

Response to the editor’s comments

| Reviewer-I | | | |
| --- | --- | --- | --- |
| No. | Editor’s comments | Author’s response | References |
|  | Thank you for inviting me for reviewing this manuscript. The authors conducted a longitudinal study for investigating the gender differences in the association between obesity, disability, and workplace absenteeism in the Australian working population. A large recruited sample size as well as its longitudinal nature are the strength of this study. However, there are a number of concerns threatening the validity of the study results. | The authors express their gratitude to the reviewer for the time and effort. All the comments we received on this study have been taken into account in improving the quality of the article, and we present our reply to each of them separately below. | Not Applicable (N/A) |
| 1. | Please rearrange the introduction (attached document)  Generally, the introduction section is too long to follow (more than 900 words) and needs reducing the number of utilized words. | Thank you very much for your suggestion. The text has been tightened up significantly. Please have a look at the Introduction section in the track change version of the manuscript. The number of words in this section has been reduced to 623 from over 900 words. | Please refer to the Introduction section |
| 2. | I would consider to bring statements from the line 75 to show this is a global issue and the literature at global level. You could reduce some literature which are not going to be looked at in the current study.  Then narrow down and introduce the Australian concept, what are the problems, what was found was found and the gap. | Thank you very much for your advice. The introduction section has been revised according to the suggestion. In doing so, the literature at the global level has been introduced and then narrowed down to the Australian context in order to identify gaps in the research. | Marked with red text in the paper. Please refer to the Introduction section, page 4, lines 3 to 15. |
| 3. | One of the main focus of this study is the Gender difference (in the Title). However, this is difficult to find where it was first reported. It is possibly first time in line 261. It could be introduced earlier, possibly after the outcome variable. | Thank you very much for raising the issue. A subsection on gender difference has been added after the description of the outcome variable in the manuscript. | Marked with red text in the paper. Please refer to the gender difference subsection, page 7, lines 14 to 23. |
| 4. | Some results are discussed in the results section which should go to the discussion. Some statements related to materials and methods are in the results section. Some results are presented with too details or too descriptive whereas those are presented in Tables and Figures. I would consider to present the key findings and refer the rest to the Tables/Figures. | Thank you very much for your comments and suggestion. The authors revised and restructured the manuscript accordingly. The result section has been tightened up and statements related to materials and methods are now moved from the results to methods section. Please have a look at the result section in the track change version of the manuscript. | N/A |
| 5. | Absenteeism is too skewed. Please present with median and range. | Thank you very much for your suggestion. Median and range values have been reported in Table 1. | Marked with red text in the paper. Please refer to the result section, page 11, lines 19 to 21. |
| 6. | Although gender differences are the major focus, it was not presented in the methods section. | Thank you very much for raising the issue. A subsection on gender difference has been added in the methods section after the description of the outcome variable in the revised manuscript.  Work and health-related behaviours often differ by gender [1]. The existing evidence reported mixed results while explaining the association between obesity and absenteeism [2]. The inconsistent findings may be due to variables that moderate the relationship. A previous study identified the variable, gender, as moderating the association between job-related factors and workplace absenteeism [3]. Attendance rate is an avenue in which women differ from men in the workplace [4]. Keeping this in mind, the present study is conducting gender-specific analyses while examining the longitudinal association between obesity, disability, and absenteeism. Moreover, this study includes a multiplicative interaction term, BMI × gender, in the regression model to test whether the joint effect of BMI and gender is significant in explaining workplace absenteeism. | Marked with red text in the paper. Please refer to the gender difference subsection, page 7, lines 14 to 23. |
| 7. | Please report what are the models 1, 2, 3 and 4 are (covariate-adjusted) and present only Model 1 and Model 2. If there are no changes in models after additional covariate adjustment, no points to show the Models. It can be said in the text that this………….was done but the model did not change. Even, Model 1 can be presented in the text and Model 2 can be presented in Table 2 for Total and by Gender by combining Table 2, 3, and 4. This will give side by side comparison without losing any information. | Thank you very much for your suggestion. The authors took into account all the suggestions made by the reviewer by merging three tables into Table 2. One thing needs to be mentioned here that the analyses have been revised in that obese categories were merged into a single variable, obesity, and the interaction of BMI and disability has been omitted as it was not significant. | Please refer to Table 2. |
| 8. | Abstract  Please don’t use the abbreviation when using IRR for the first time. | The full form of IRR has been mentioned in the text. | Please see the red text in the abstract section, page 2, line 14 |
| 9. | Line 41: Please clearly determine which type of interaction i.e. additive or multiplicative has not been significant? | Thank you very much for raising the point. In the text, the type of interaction has been mentioned clearly.  This study found multiplicative interaction between weight status and gender is significantly associated with absenteeism. | Please see the red text in the abstract section, page 2, line 17-18 |
| 10. | Line 44: Conclusions: what about male workers? | In the earlier version of the manuscript, the author focused on workplace health promotion programs only for overweight and obese women workers. However, the authors do agree that workplace health promotion programs should be implemented irrespective of gender. Hence, the text has been changed to remove the ambiguity.  Workplace absenteeism is significantly associated with overweight and obesity among Australian workers. An active workplace health promotion program is a necessity for weight management of overweight and obese workers and thus reduced workplace absenteeism. For example, employers may provide incentives for maintaining the recommended body weight, encourage exercise, and promote healthy diets. | Please see the red text in the abstract section, page 3, line 3-7 |
| 11. | METHOD Line 128-130: The different employed method for obtaining the study information i.e. a combination of personal face-to-face interviews by trained interviewers and self-completed questionnaires might impose an information bias to the study findings. Please clarify this discrepancy. | Thank you very much for your suggestion. A clarification has been provided in the text.  It collects detailed information from household members aged 15 years and over using a combination of face-to-face interviews and telephone interviews by trained interviewers, and self-completed questionnaires. There might have a concern that responses collected through different modes have a significant impact on the data. However, preliminary findings suggest that there is little systematic bias in responses by data collection modes [5]. | Please see the red text in the Method section, page 6, line 10-14 |
| 12. | Line 135-136: what do you mind by “prospective subjects” in the following, sentences: “The inclusion criteria of the study are prospective subjects aged 15-64 years and employed at each wave.” | Thank you very much for the query. The text has been changed to dispel the ambiguity.  The inclusion criteria of the present study are participants aged between 15 and 64 years and employed at each wave. | Please see the red text in the Method section, page 6, line 17-18 |
| 13. | Line 138: please clarify for your readers why pregnant employees with a large sample size (n = 8631 observations) were excluded from the subsample analyses. | Clarification has been given in the text now.  Further, pregnant employees were excluded (n = 8631 observations) from the subsample analyses to avoid potential bias and ensuring the validity of the study findings. | Please see the red text in the Method section, page 6, line 20-22 |
| 14. | Line 148-149: Although the authors stated the self-reported nature of BMI in the limitation section, however, the self-reported BMI as well as disability could imposed an important information bias to the study finding. | Thank you very much for raising the issue. A detailed explanation has been given in the limitation part of the manuscript.  Second, the study findings might be vulnerable to bias, as data on BMI, disability, and absenteeism are self-reported. Self-reported bias is high among overweight and obese adults, as they tend to overestimate their height and underestimate their weight [6,7]. Similarly, there might be justification bias in case of self-reported disability as individuals tended to over-report their disability level for financial benefits that can accrue [8]. | Please see the red text in the Conclusion section, page 19, line 18-24 to page 20, line 1-2 |
| 15. | Line 164: what was the utilized criteria for selecting potential confounder? | The criteria used for selecting the confounders have been included in the manuscript.  This study selected potential confounders following relevant published studies on the risk factors of workplace absenteeism [2,9–20] and information available in the HILDA datasets. Confounders were included in the fully adjusted model only if a confounder was found significant at 5% or less risk level at any level in the bivariate analyses. | Please see the red text in other covariates section, page 8, line 19-22 |
| 16. | Line 166: What about the role of sex (male and female)? Is it confounder or effect modifier? | Thank you very much for the query. The variable ‘gender’ serves as a confounder. | N/A |
| 17. | There is not information about the potential confounding role of the other comorbidities. | Thank you very much for raising the issue. Information regarding the confounding role of the other comorbidities has been included in the text.  Confounding role of other comorbidities such as cancer, diabetes, heart disease, depression, asthma, bronchitis, and arthritis in explaining workplace absenteeism could not be explored in the present study. The principal reason for not exploring such roles is that these information were available only in waves 9, 13 and 17 of the HILDA survey. | Please see the red text in other covariates section, page 9, line 11-14 |
| 18. | Please explain the goodness of fit criteria and model-fitting process for 4 introduced models. | Thank you very much for raising the issue. Information regarding the goodness of fit criteria and the model-fitting process has been included in the text.  To find out the longitudinal association between the exposures and outcome variable, the present study followed forward addition approach for building model. In this approach, the multivariate model starts with the basic model where BMI is the exposure and absenteeism is the outcome variable. Confounders and interaction terms were added one at a time based on its level of significance. The process continued until all significant confounders and interaction term were included in the model. | Please see the red text in Methods section, page 10, line 5-10 |
| 19. | The results show that obese class I and II were significantly associated with absenteeism but not with overweight and obese class III. Please explain this inconsistent finding? | Thank you very much for the comment. The inconsistent findings have been removed from the results. The analyses have been revised in that obese categories were merged into a single variable, obesity, and the interaction of BMI and disability has been omitted as it was not significant. In the updated results section there is no inconsistent findings. | Please refer to Table 2. |
| 20. | Please revise the reported interpretation of IRR throughout the manuscript. Please note that the RATE measures the rate of the occurrence of the study event (here, absenteeism) not its probability. For example when interpreting the rate of obese class II workers (2.18 (IRR: 2.18, 95% CI: 1.43-3.33)) with their lower weight counterparts, the IRR tells the rate of absenteeism in obese class II workers compared with the lower weight. (Similar interpretation has reported in lines: 256, 257, …) | Thank you very much for your suggestion. The interpretations have been revised throughout the manuscript.  The findings indicate that the rate of workplace absenteeism in overweight and obese workers were 1.24 (IRR: 1.24, 95% CI: 1.03-1.49) and 1.46 (IRR: 1.46, 95% CI: 1.20-1.78) times higher compared with their lower weight counterparts, respectively. Model 1 also revealed that the number of days absent from the workplace among workers with a disability was 3.02 times (IRR: 3.02, 95% CI: 2.50-3.64) higher compared with workers without a disability. Model 2 reports a significant association between the interaction of BMI and gender with prolonged absenteeism. The results showed that the rate of absenteeism was 2.52 times (IRR: 2.52, 95% CI: 1.75-3.63) and 1.65 times (IRR: 1.65, 95% CI: 1.13-2.40) higher among overweight and obese women employees than their male counterparts, respectively. | Marked with red text in the paper. Please refer to the results section, page 14, line 03-24. |
|  | Line 258: please state which type of interaction; additive or multiplicative has not been identified between obesity and disability and prolonged absenteeism. | Thank you very much for your suggestion. Now it has been mentioned in the text.  The present study also found significant multiplicative interaction of BMI and gender in explaining workplace absenteeism. | Marked with red text in the paper. Please refer to the discussion section, page 17, line 19-20. |
| 21. | Please clarify the sample size and its possible over-power. | Thank you very much for your suggestion. Clarification has been provided in the text.  After employing inclusion criteria and excluding missing data, the unbalanced panel consists of 107,669 observations from 19,174 adult employees. Study participants were generated from the HILDA dataset following the HILDA survey protocol. HILDA uses a multi-stage sampling approach, as well as sampling within households within a particular administrative area. Detailed information about the sampling procedure and design have been described elsewhere [21].  The method section has been revised as follows:  The authors constructed an unbalanced longitudinal data set consisting of 107,669 observations by linking 19,174 individuals’ records who participated in either of the waves from 6 to 17 of the HILDA survey. | Marked with red text in the paper. Please refer to the sample section, page 6, lines 22-24 to page 7 line 1-3.  Further, please refer to the method section, page 9, line 16-18. |
| 22. | Please test the possible interaction between sex and overweight in absenteeism. | Thank you very much for your suggestion. Interaction between sex and overweight in absenteeism has been tested and reported in Table 2. | Please refer to table 2 (model 2). |
| 23. | Table 1  What is 8.70 in the first row? Is it SD? If yes, please explain why it is very larger than its Mean? Please separately report the mean (SD) in non-zeros. | Thank you very much for the query. Yes, it is SD. The explanation has been provided in the text. As suggested, mean (SD) in non-zeros are also reported in Table 1.  A higher value of the standard deviation over mean indicates that the absent days variable is over dispersed with excessive zeros. | Marked with red text in the paper. Please refer to the result section, page 11, line 19-21.  Further, please refer to Table 1. |
| 24. | While obese class II has shown a significant association with absenteeism, obese class III has not shown such an association. Please explain? | Thank you very much for your query. The inconsistent findings were removed from the results. The analyses have been revised in that obese categories were merged into a single variable, obesity, and interaction of BMI and disability has been omitted as it was not significant. In the updated results section there is no inconsistent finding. Please refer to Table 2. | Please refer to Table 2 |
| 25. | Please use “confounder” throughout the manuscript instead of control variables. | Thank you very much for your suggestion. The word ‘confounder’ has been used throughout the manuscript. | Please see the red text in other covariates section, page 8, line 19-22 |
| 26. | Obese class III (≥40.00) has shown a significant association only in model I but not in the other models. Please explain. | As stated above, the analyses have been revised in that obese categories were merged into a single variable, obesity. In the updated result there is no inconsistent finding. | Please refer to Table 2 |
| 27. | Table 4  An unexpected point estimates of potential interactions in table 4 is reported for possible interaction between Obesity and disability. The reported point estimates are protective! Please explain. | As stated above, the inconsistent findings were removed from the results. The analyses have been revised in that obese categories were merged into a single variable, obesity, and interaction of BMI and disability has been omitted as it was not significant. In the updated results section no inconsistent findings are reported. | Please refer to Table 2 |
| 28. | Some reported numbers in Table 4 is without decimal numbers. Please keep a unique scheme for representing the numbers. | Thank you very much for your suggestion.  Uniform decimal numbers have been ensured in Table 1 and Table 2. | Please refer to Table 1 and Table 2. |
| 29. | The point estimates for interaction terms in Table 3 are surprising. While the interaction terms between Obese class I and having disability is larger than 1, this is protective for Obese class II and having disability! | As stated above, the analyses have been revised in that obese categories were merged into a single variable, obesity, and interaction of BMI and disability has been omitted as it was not significant. In the updated result there is no inconsistent finding. | Please refer to Table 2. |
| 30. | There is no information regarding the percent of lost to follow up and its potential impact on the study findings. | Thank you very much for the query. The information regarding the percent of lost to follow up and its potential impact on the study findings has been included in the text.  The percentage of participants who were lost due to missing information on the outcome variable and pregnancy was 1.99% and 7.27%, respectively. The total percentage of loss to follow-up in the present study is less than 10% which seems common for longitudinal studies and thus leads to little bias. | Marked with red text in the paper. Please refer to the sample section, page 7, line 4-7. |
| 31. | Line 300: Please explain the reported discrepancy between the significant interactions between the study variables in male but not females? | Thank you very much for asking for clarification. The clarification has been mentioned based on current findings in the manuscript.  An important cause of this gender difference in workplace absenteeism may be the menstrual cycle [22]. Further, the gender difference in absenteeism could be attributed to women’s double burden of wage work and unpaid household chores [23]. Another possible explanation is women typically perform more monotonous and stressful jobs [23]. | Marked with red text in the paper. Please refer to the discussion section, page 18, line 3-7. |
| 32. | CONCLUSSION With the study limitations as well as | Thank you very much for your suggestion. Limitations of the study have been presented in the conclusion section as follows:  The present study has some limitations. First, the unbalanced longitudinal design of the study draws longitudinal associations but it is not possible to discern the causal effect of obesity, and disability on workplace absenteeism. Second, the study findings might be vulnerable to bias, as data on BMI, disability, and absenteeism are self-reported. Self-reported bias is high among overweight and obese adults, as they tend to overestimate their height and underestimate their weight [6,7]. Similarly, there might be justification bias in case of self-reported disability as individuals tended to over-report their disability level for the financial benefit [8]. The authors call for a well-designed cohort study that can draw causal inferences on the association between obesity, disability, and absenteeism. | Marked with red text in the paper. Please refer to the conclusion section, page 19, line 18-24 to page 20, line 1-2. |

**References**

1.	Sendén MG, Schenck-Gustafsson K, Fridner A. Gender differences in Reasons for Sickness Presenteeism - a study among GPs in a Swedish health care organization. Ann Occup Environ Med. Annals of Occupational and Environmental Medicine; 2016;28: 1–7. doi:10.1186/s40557-016-0136-x

2.	Harvey SB, Glozier N, Carlton O, Mykletun A, Henderson M, Hotopf M, et al. Obesity and sickness absence: Results from the CHAP study. Occup Med (Chic Ill). 2010;60: 362–368. doi:10.1093/occmed/kqq031

3.	Scott KD, Mabes DA. The Job Satisfaclion/Absenteeism Relationship: Gender as a Moderating Variable. Akron Bus Econ Rev. 1984;15: 43–47.

4.	Scott KD, McClellan EL. Gender Differences in Absenteeism. Public Pers Manage. 1990;19: 229–254. doi:10.1177/009102609001900210

5.	Watson N, Wooden M. Mixed and multiple collection modes: The HILDA Survey experience. paper presented to XXV International Methodology Symposium-Longitudinal Surveys: From Design to Analysis. Statistics Canada, Ottawa-Gatineau, 27-30 October;

6.	Maukonen M, Männistö S, Tolonen H. A comparison of measured versus self-reported anthropometrics for assessing obesity in adults: a literature review. Scand J Public Health. 2018;46: 565–579. doi:10.1177/1403494818761971

7.	Gorber SC, Tremblay M, Moher D, Gorber B. A comparison of direct vs. self-report measures for assessing height, weight and body mass index: A systematic review. Obes Rev. 2007;8: 307–326. doi:10.1111/j.1467-789X.2007.00347.x

8.	Black N, Johnston DW, Suziedelyte A. Justification bias in self-reported disability: New evidence from panel data. J Health Econ. Elsevier B.V.; 2017;54: 124–134. doi:10.1016/j.jhealeco.2017.05.001

9.	Fitzgerald S, Kirby A, Murphy A, Geaney F. Obesity, diet quality and absenteeism in a working population. Public Health Nutr. 2016;19: 3287–3295. doi:10.1017/S1368980016001269

10.	Frone MR. Obesity and absenteeism among U.S. workers: Do physical health and mental health explain the relation? J Workplace Behav Health. 2007;22: 65–79. doi:10.1080/15555240802157403

11.	Howard JT, Potter LB. An assessment of the relationships between overweight, obesity, related chronic health conditions and worker absenteeism. Obes Res Clin Pract. Asia Oceania Assoc. for the Study of Obesity; 2014;8: e1–e15. doi:10.1016/j.orcp.2012.09.002

12.	Roos E, Laaksonen M, Rahkonen O, Lahelma E, Lallukka T. Weight change and sickness absence - A prospective study among middle-aged employees. Eur J Public Health. 2015;25: 263–267. doi:10.1093/eurpub/cku087

13.	Vanwormer JJ, Linde JA, Harnack LJ, Stovitz SD, Jeffery RW. Weight change and workplace absenteeism in the healthworks study. Obes Facts. 2012;5: 745–752. doi:10.1159/000345119

14.	Jans MP, Van Den Heuvel SG, Hildebrandt VH, Bongers PM. Overweight and obesity as predictors of absenteeism in the working population of the Netherlands. J Occup Environ Med. 2007;49: 975–980. doi:10.1097/JOM.0b013e31814b2eb7

15.	Ferrie JE, Head J, Shipley MJ, Vahtera J, Marmot MG, Kivimäki M. BMI, obesity, and sickness absence in the whitehall II study. Obesity. 2007;15: 1554–1564. doi:10.1038/oby.2007.184

16.	Nigatu YT, Roelen CAM, Reijneveld SA, Bültmann U. Overweight and distress have a joint association with long-term sickness absence among Dutch employees. J Occup Environ Med. 2015;57: 52–57. doi:10.1097/JOM.0000000000000273

17.	Lehnert T, Stuhldreher N, Streltchenia P, Riedel-Heller SG, König HH. Sick leave days and costs associated with overweight and obesity in germany. J Occup Environ Med. 2014;56: 20–27. doi:10.1097/JOM.0000000000000065

18.	Janssens H, Clays E, Kittel F, De Bacquer D, Casini A, Braeckman L. The association between body mass index class, sickness absence, and presenteeism. J Occup Environ Med. 2012;54: 604–609. doi:10.1097/JOM.0b013e31824b2133

19.	Tucker LA, Friedman GM. Obesity and absenteeism: An epidemiologic study of 10,825 employed adults. Am J Heal Promot. 1998;12: 202–207. doi:10.4278/0890-1171-12.3.202

20.	Reber KC, König HH, Hajek A. Obesity and sickness absence: Results from a longitudinal nationally representative sample from Germany. BMJ Open. 2018;8: 1–8. doi:10.1136/bmjopen-2017-019839

21.	Freidin, S., Watson, N. and Wooden M. Household, Income and Labour Dynamics in Australia (HILDA) Survey: Wave 1. Melbourne; 2002.

22.	Ichino A, Moretti E. Biological gender differences, absenteeism, and the earnings gap. Am Econ J Appl Econ. 2009;1: 183–218. doi:10.1257/app.1.1.183

23.	Karlsson T. Gender differences in absence from work : Lessons from two world wars. Work Pap. 2016;2016.
