# Supplementary material for: Gender differences in the longitudinal association between obesity, and disability with workplace absenteeism in the Australian working population
Source: PLoS One. 2020 May 27;15(5):e0233512. doi: 10.1371/journal.pone.0233512 (PMC7252611; doi:10.1371/journal.pone.0233512)
Supplement: Attachment — Submitted filename: Response to the Reviewers-1.docx [file pone.0233512.s003.docx]

Response to the editor’s comments

| Reviewer-I | | | |
| --- | --- | --- | --- |
| No. | Editor’s comments | Author’s response | References |
|  | A significant improvement, would like to suggest dealing some minor issues | Thank you very much for the appreciation. The authors are grateful to the reviewer for the wonderful suggestions and advice to improve the quality of the manuscript. All the comments we received so far on this study have been taken into account in improving the quality of the article, and we present our reply to each of them separately below. | Not Applicable (N/A) |
| 1. | Table 1: Data seems mean (95%CI), however, the title “Mean (SD)/ % (CI)” seems confusing. May be something could be presented referring a footnote.  Numbers are large, once decimal place would look better. I mean, instead of 66.38 (66.09 -66.66), 66.4 (66.1-66.7) throughout would look better. | Thank you very much for the suggestion. The heading “mean (95% CI)” in Table 1 has been rectified to avoid confusion. As advised, one decimal have been applied throughout Table 1. | Please refer to the Table 1, Page 11-13 in the manuscript. |
| 2. | Table 2: what is the difference between Model 1 and Model 2 (total sample). This is assumed, one is unadjusted and the other is multivariate-adjusted. This has not been reported. What covariates were in the Model is not known here?. * indicates significant, ** means more significant, *** very significant. In fact, all are significant. The confidence intervals automatically show which are significant and which are not. Therefore, the symbols are not necessary. I would suggest presenting like this,  Model 1 (total sample) IRR (95% CI)* Model 2 (total sample) IRR (95% CI)** Model 3 (only men) IRR (95% CI)**, Model 4 (only women) IRR (95% CI)**  *IRR(95%CI): Unadjusted model; **IRR (95%CI): adjusted for X,y,Z…………… If the authors are still interested to show the significance with a symbol, only one symbol could be shown and said this is for p<0.05. | Thank you very much for the advice. Table 2 has been revised following the reviewer’s advice. | Please refer to the Table 2, Page 16-18 in the manuscript. |
| 3. | Table 2: Age categories do not seem logical at all. 15-35, before adult (15-17), early adult (18-25), and adult (26-35) in one group. 30 to 35 or 36 to 45 do not seem any different in nature. There could be references when there is an age group transition. Possibly, 15-25, 26-45, 46-60, 60+. I would consider some interesting findings could come up from this large sample. Could be checked for this variable only. | Thank you very much for focusing on the issue. The variable ‘age’ has been categorized according reviewer’s advice and plugging into the adjusted model. | Please refer to the Table 2, Page 16-18 in the manuscript. |
| 4. | The variable, Overall job satisfaction (from 0 = worst to 10 = best). What does the non-significant protective association tell? As the satisfaction level increases by one point from 0 to 1 or 1 to 2, workplace absenteeism decreases a bit. This means, as the satisfaction level increases, less the absenteeism. There could have a threshold cut-off or binary cut-off where it could be significantly higher. I would suggest checking this variable if there is any binary cut-off where it could be significant | Thank you very much for your comments and suggestion. The authors have categorized the variable ‘overall job satisfaction’ and plugging into the adjusted model as confounders according to the reviewer’s suggestion. | Please refer to the Table 2, Page 16-18 in the manuscript. |
